# Neurobehavioral Effects of Cephalosporins: Assessment of Locomotors Activity, Motor and Sensory Development in Zebrafish

**DOI:** 10.3389/fphar.2018.00160

**Published:** 2018-03-02

**Authors:** Ying Han, Yangmin Zheng, Jingpu Zhang, Changqin Hu

**Affiliations:** ^1^Division of Antibiotics, National Institutes for Food and Drug Control, Beijing, China; ^2^Department of Pharmacology, Institute of Medicinal Biotechnology, Chinese Academy of Medical Sciences and Peking Union Medical College, Beijing, China

**Keywords:** neurobehavioral effects, zebrafish, transcriptomics, impurities, docking

## Abstract

Most third- and fourth-generation cephalosporins, such as cefotaxime, cefmenoxime, cefepime, and cefpirome, contain an aminothiazoyl ring at the C-7 position. Drug impurity, which may be produced either during synthesis or upon degradation, can induce adverse effects. Various reports have indicated that neurotoxicity is a side effect of cephalosporin. In this study, we developed methods for assessing the free-swimming activities and behaviors in zebrafish larvae in response to continuous darkness and stimulation of light-to-dark photoperiod transition by chemical treatments. We also performed transcriptome analysis to identify differentially expressed genes (DEGs). Gene ontology analysis revealed that various processes related to nervous system development were significantly enriched by DEGs. We integrated 16 DEGs with protein–protein interaction networks and identified that neuroactive ligand–receptor interaction [e.g., λ-aminobutyric acid and glutamate receptor, metabotropic 1a (GRM1A)] pathway was regulated by the compounds. Our findings suggested that neurobehavioral effects mainly depend on the mother nucleus structure 7-aminocephalosporanic acid and the substitution at the C-3 position. In addition, *gad2*, *or111-4*, *or126-3*, *grm1a*, *opn8c*, *or111-5*, *or113-2*, and *or118-3* may potentially be utilized as novel biomarkers for this class of cephalosporins, which causes neurotoxicity. This study provides neurological behavior, transcriptome, and docking information that could be used in further investigations of the structures and developmental neurotoxicity relationship of chemicals.

## Introduction

**FIGURE 1:**
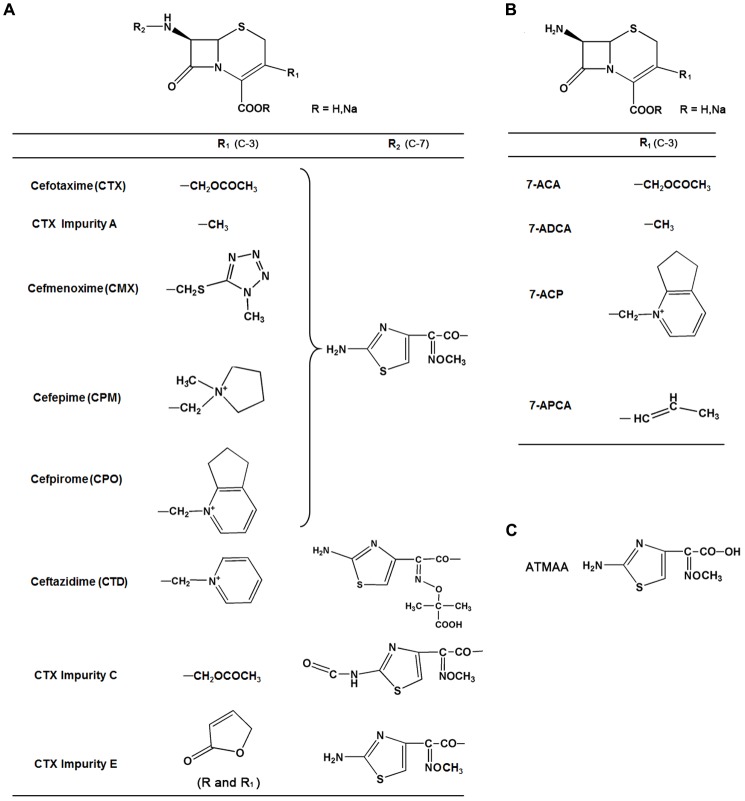
Chemical structures, common names, and abbreviations of 13 compounds investigated in this study. **(A)** Group I. **(B)** Group II. **(C)** ATMAA.

Cephalosporin, a broad-spectrum antimicrobial with low toxicity and resistance to penicillin enzyme, accounts for about 50% antibiotic sales in China. Most of the third- and fourth-generation cephalosporins, such as cefotaxime (CTX), cefmenoxime (CMX), cefepime (CPM), and cefpirome (CPO), consist of an acetyl side chain with an aminothiazoyl ring (**Figure 1**). CTX sodium, a third-generation cephalosporin antibiotic, is a semisynthetic cephalosporin; it has the structure of an aminothiazoyl ring with a *syn* methoxyimino residue at C-7 position, and the C-3 side chain is an acetoxymethyl moiety. It has broad-spectrum antimicrobial activity and still has a wide market at present. Various reports have indicated that neurotoxicity is a side effect of cephalosporin. Cephalosporin may cause a wide spectrum of neurologic effects, including altered mentation, myoclonus, asterixis, coma, seizures, and status epilepticus; the frequency or incidence of these effects is not known (Grill and Maganti, 2008). The most frequent neurotoxicity reports are seen with first-generation cephalosporins such as cefazolin, second-generation cephalosporins such as cefuroxime, third-generation cephalosporins such as ceftazidime (CTD), and fourth-generation cephalosporins such as CPM (Grill and Maganti, 2011). Previous reports showed that CPM neurotoxicity could appear in patients with renal impairment, and most cases have occurred in patients whose standard dosages were not adjusted for their renal function (Garces et al., 2008; Fugate et al., 2013). However, some articles also reported that CPM induced neurotoxicity in patients with normal renal function (Coelho et al., 2010; Meillier and Rahimian, 2016). The main mechanism of cephalosporin neurotoxicity involves a decrease of λ-aminobutyric acid (GABA) released from nerve terminals and a subsequent increase of excitatory neurotransmission. The potential neurotoxicity mechanisms and the relationship between the neurotoxicity and structure of this type of cephalosporin are still unclear.

Impurities in a drug product are considered to be one of the main factors in drug safety problems (Alsante et al., 2014). Recently, an impurity profile has become a part of the regulatory requirements for drugs. The International Conference on Harmonization (ICH) has published guidelines for the control of impurities in drug substances and products. The drug product registry in China [i.e., the Chinese Food and Drug Administration (CFDA)] and those in many other countries follow the ICH guidelines to control the quality and safety of drugs. Impurity may be produced either during the synthetization process or upon degradation in medicines. When the content of impurities in drugs is greater than the qualification threshold for chemical medicine (0.15%), a toxicity assessment of impurities should be performed (ICH, 2006). Ideally, quality control of the impurity profile should be based on the physiological activity of each impurity in the drug (Görög, 2006).

The structure–activity relationship of cephalosporin side chains has been extensively explored. However, only a few reports have described the structure–toxicity relationship of cephalosporins in detail, especially the toxicity of their impurities. In order to study structure–toxicity relationship of drugs, control the quality and safety of drugs, and speed up the drug development processes, we previously established the zebrafish embryo toxicity models (Zhang et al., 2015; Han et al., 2017; Qian et al., 2017), cardiac toxicity models (Han et al., 2015), and neurotoxicity models (Chen et al., 2017) for the evaluation of drug toxicity, containing the active pharmaceutical ingredients and impurities’ toxicity assessment. Our previous studies on zebrafish embryo toxicity testing also suggested that both the C-7 and C-3 substituents of cephalosporins are toxic functional groups (Zhang et al., 2013; Han et al., 2017), and the C-3 side chain of cefoperazone may induce bleeding (Han et al., 2017). The small size, transparency, and high fecundity of the zebrafish embryo make it easy to observe the development of organs and tissues with a simple optical microscope. The brain of the zebrafish is simple, but also contains the telencephalon, diencephalon, midbrain, and hindbrain. At 5-days post-fertilization (dpf), zebrafish larvae show spontaneous swimming and their visual system is fully developed. As many reports suggest that the behavioral responses of zebrafish are similar to those of mammals (Noyes et al., 2015; Demin et al., 2017; Kim et al., 2017), it seems appropriate to use zebrafish for testing of exercise, learning, and memory in behavioral evaluation.

In this study, we investigated the effects of cephalosporin and impurities on behavior in zebrafish larvae, and the resulting embryo transcriptomes were compared in order to identify the biomarkers for neurotoxicity. We demonstrated that exposure to compounds altered the expression of several genes that are critical for normal function of the nervous system. Overall, our study provided insights on transcriptomic changes that correspond to behavior alterations in response to exposure to compounds and a better understanding of the relationship between the structure and toxicity mechanisms of cephalosporins.

## Materials and Methods

### Laboratory Animals

Zebrafish (*Danio rerio*) of the TU wild-type strain were originally obtained from the Institute of Medicinal Biotechnology, Chinese Academy of Medical Sciences, and Peking Union Medical College (Beijing, China). The zebrafish were normally maintained under a 14-h light/10-h dark cycle in an automatic circulating tank system and fed live brine shrimp once daily. The water temperature was maintained at 28 ± 1°C and pH 7.0 ± 0.5. The day before spawning, two pairs of adult zebrafish were placed in a breeding tank equipped with a spawning tray. Shortly after spawning, embryos were collected from the tank and placed in Petri dishes filled with embryo water (60 mg L^-1^ Instant Ocean salts, pH 7.2) (Westerfield, 2007), and fertilized embryos were selected for all experiments. All the experimental protocols were approved by the Committee on the Ethics of Animal Experiments of the Institute of Medicinal Biotechnology, Chinese Academy of Medical Sciences (IMBF20060302), which is in accordance with the NIH Guidelines for the Care and Use of Laboratory Animals^1^.

### Chemicals

The reference standards of CTX sodium, CMX hydrochloride, CPM, CPO sulfate, CTD, 7-amino-3-methyl-8-oxo-5-thia-1-azabicyclo[4.2.0]oct-2-ene-2-carboxylic acid (7-ADCA), (6R, 7R)-7-amino-3-(6,7-dihydro-5H-cyclopenta[b]pyridin-1-ium-1-ylmethyl)-8-oxo-5-thia-1-azabicyclo[4.2.0]oct-2-ene-2-carboxy-late (7-ACP), 7-aminocephalosporanic acid (7-ACA), and (6R,7R)-7-amino-8-oxo-3-[(E)-prop-1-enyl]-5-thia-1-azabicyclo[4.2.0]oct-2-ene-2-carboxylic acid (7-APCA) were obtained from the National Institutes for Food and Drug Control (Beijing, China). CTX impurity A, impurity C, impurity E, and 2-(2-aminothiazole-4-yl)-2-methoxyiminoacetic acid (ATMAA) were provided by China Resources Shenzhen Gosun Pharmaceutical, Co., Ltd. (Shenzhen, China). The structure of each API and impurity was confirmed by MS and NMR; the purity of API (>95%) and impurity (>93%) were normalized by HPLC or by NMR.

### Behavioral Testing

According to our previously study (Chen et al., 2017), 30 successfully hatched larvae (5 dpf) were selected and placed according to their treatment groups. All larvae were placed in 2-cm Petri dishes in a 28 ± 1°C incubator. After 1 day of exposure, eight individual fish with no obvious malformations were transferred to 48-well plates, with each well-containing a single fish larva. The control group was untreated larvae. Behavioral testing was begun on 6 dpf at 13:00. The locomotor activity was measured by the ZebraLab Video-Track system version 3.3 (ViewPoint Life Science, France), as the activity was relatively stable. The tests were under specific conditions: after 10 min of acclimation in the dark, (1) free swimming activities were detected and recorded in continuous darkness (20 min), and (2) the larvae were exposed to three cycles of alternating 10-s light and 5-min dark photoperiod stimulation periods. The movement distance and speed were observed. Speed less than 0.2 cm/s was defined as inactive motion; speed greater than 0.2 cm/s was defined as active motion.

### Microarray Scanning

Embryos at 6 hpf exposed to 15-mM compounds and collected at 10 hpf for microarray. The treatment concentration was according to the lethal concentration 50 of compounds (data not shown). The control group was untreated larvae. The Agilent zebrafish (V3) gene expression microarray 4 × 44K (Design ID 026437), obtained from CapitalBio Corporation (Beijing, China), was used for microarray analysis. Samples consisting of 100 embryos per compound were snap frozen in liquid nitrogen. Total RNA was extracted using TAKARA RNAiso Plus following the manufacturer’s instructions, and its RIN number was detected using an Agilent Bioanalyzer 2100 (Agilent Technologies, Santa Clara, CA, United States) to determine RNA integrity. Qualified total RNA was further purified using an RNeasy Mini Kit (QIAGEN, GmBH, Germany) and an RNase-free DNase Set (QIAGEN).

Total RNA was amplified and labeled using a Low Input Quick Amp Labeling Kit, One-Color (Agilent Technologies), following the manufacturer’s instructions. Labeled cRNA was purified using an RNeasy mini kit (QIAGEN). Each slide was hybridized with 1.65 μg of Cy3-labeled cRNA using a gene expression hybridization kit (Agilent Technologies) in a hybridization oven (Agilent Technologies) according to the manufacturer’s instructions. After 17 h of hybridization, the slides were washed in staining dishes (Thermo Shandon, Waltham, MA, United States) with a gene expression wash buffer kit (Agilent Technologies), following the manufacturer’s instructions. The slides were scanned on an Agilent Microarray Scanner (Agilent Technologies) using default settings, namely, green dye channel, scan resolution = 5 μm, PMT 100%, 10%, 16-bit. Data were extracted with Feature Extraction software version 10.7 (Agilent Technologies). Raw data were normalized using Quantile Algorithm, GeneSpring Software 12.6.1 (Agilent Technologies).

### Microarray Data, GO, and Pathway Enrichment Analyses

To identify differentially expressed genes (DEGs) between the control and compound groups, the threshold of fold-change (linear) ≥ 3 was established in our analysis. To explore the implication of the identified DEGs, functional enrichment analysis of GO terms and Kyoto Encyclopedia of Genes and Genomes (KEGG) pathways were conducted using the Database for KEGG Orthology-Based Annotation System (KOBAS 3.0 Peking University, Beijing, China). Significantly enriched GO terms and pathways were determined using the hypergeometric test/Fisher’s exact test, with a threshold of *p*-values < 0.05 that were adjusted using the Benjamini–Hochberg FDR correction and with a minimum of three enriched genes considered. To determine the overlaps of significantly DEGs between compounds, we created Venn diagrams using Venny 2.1^2^.

### Protein–Protein Interaction (PPI) Network Analysis

To construct a protein–protein interaction (PPI) network for each list of proteins encoded by the DEGs, we imported the lists into the extensive database of already known networks and screened significant PPIs using the Biological General Repository for Interaction Datasets (BioGRID^3^) and the Search Tool for the Retrieval of Interacting Genes (STRING^4^) database. Then, we used Cytoscape 3.4.0 software to map the PPI network^5^.

### Quantitative Real-Time Polymerase Chain Reaction (qRT-PCR) Analysis

To validate the microarray data, the expression of eight genes were analyzed by qRT-PCR. Total RNA was extracted from 30 embryos from each treatment group and control group (6–10 hpf) using TRIzol (Sigma-Aldrich, St. Louis, MO, United States) according to the manufacturer’s protocol. First-strand cDNA was synthesized using oligo-dT primers and M-MLV reverse transcriptase (Promega, Madison, WI, United States) according to the manufacturer’s instructions. qRT-PCRs were performed in a ROCHE Light Cycler 96 system (Roche, Switzerland). Primers for confirmation of selected genes were designed using AlleleID 6.0 (PREMIER Biosoft, CA, United States) and synthesized by Sangon Biotech Co., Ltd. (Beijing, China). The primer sequence list is provided in **Table 1**. Relative transcript expression was determined using β-actin as reference, and data normalization was performed using the ΔΔCT comparative quantization method.

**Table 1:** Primers used in the quantitative real-time polymerase chain reaction analysis of different gene markers.

Gene	Primer F sequence (5′–3′)	Primer R sequence (5′–3′)
*or111-5*	ATCGGCACCAGTTAGAGAGG	CAGTAAATACACCCAGTTGACATC
*or111-4*	TTCTGTGGTAGCCTCTATGG	ACAAAGCACTGATGAAATAAGC
*or113-2*	CACCTGCGTCACTCACATC	TGTTTGTCTTATCTCTTTCGTTCG
*or118-3*	GCTCAACCTGCCAATCAATG	TGCGATATACCTGTCAAATGC
*opn8c*	ATCTGGCTTGAAGGAGTC	TTGTGGATGTATGGTAACG
*or126-3*	GAGAGCAGGCACAAGTTC	CACATTCTTTGACCCATAACG
*grm1a*	AAGCCTTCCTGAACCAACC	CCTCCTCGTCGTGAATCTG
*gad2*	GTGCTCTGCTCTTCTGGTTC	GCCTTGTCTCCTGTGTCATAC
*β-actin*	AGGGAAATCGTGCGTGACATCA	ACTCATCGTACTCCTGCTTGCTGA

### Data Analysis

All data were plotted by GraphPad Prism 6.0 (GraphPad Software, Inc., United States). Statistical analyses were performed using IBM SPSS software version 20.0 (IBM Corporation, Armonk, NY, United States). Swimming times and speeds were compiled as means and standard errors of means, with treatment comparisons carried out by one-factor ANOVA followed by Dunnett’s *post hoc* analysis. Significance for all tests was assumed at *p* < 0.05, *p* < 0.01, or *p* < 0.001.

### Molecules’ Preparation and Docking

The Discovery Studio 4.0 (DS 4.0) software package (Accelrys Software, Inc., San Diego, CA, United States) was used for the docking study of selected targets and ligands. For protein preparation, the 3D crystallographic structure of glutamate decarboxylase 2 (GAD2) and glutamate receptor, metabotropic 1a (GRM1A), was modeled through homology modeling server SWISS-MODEL^6^ (Arnold et al., 2006; Biasini et al., 2014). Before docking, hydrogen atoms were added to the unoccupied valence of the heavy atoms of the protein. GAD2 and GRM1A proteins were defined as a total receptor using DS 4.0. For ligand preparation, the structures of CTX, CMX, impurity A, CPO, and CPM were downloaded from the PubChem Compound Database^7^. From the receptor–ligand interaction section of DS 4.0, CDOCKER protocol was chosen to conduct the docking studies. The docking was performed with a simulated annealing method to minimize the CDOCKER energy for obtaining an optimum pose.

## Results

### Effects of CTX and Five Impurities on the Behavior of Larval Zebrafish

**FIGURE 2:**
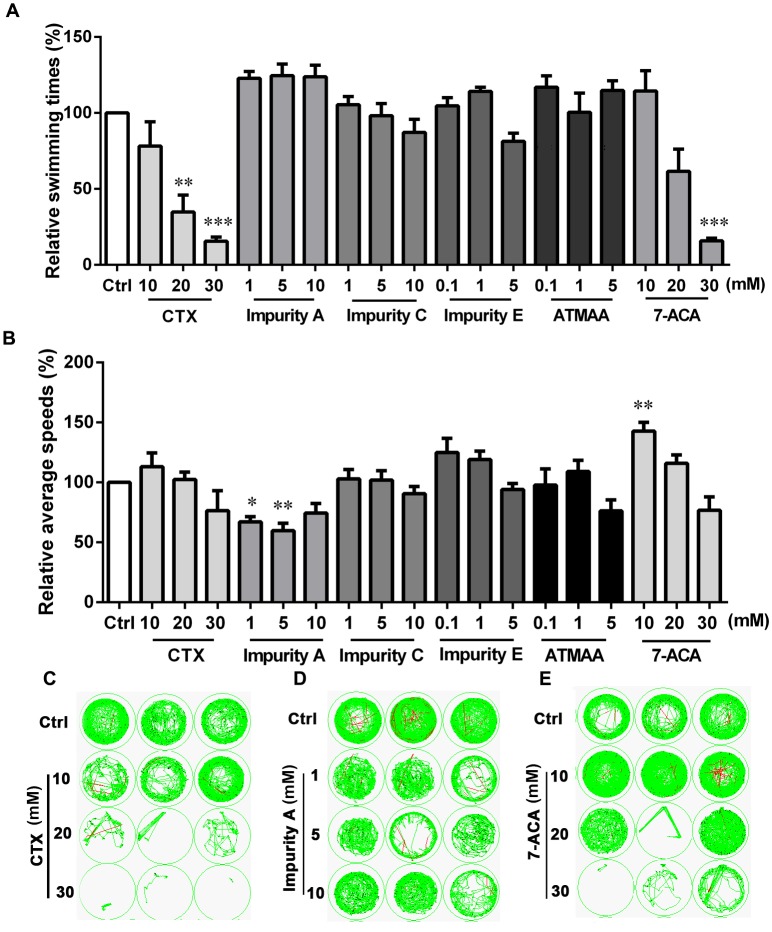
Locomotor behavior of the larval zebrafish after exposure to various concentrations of cefotaxime (CTX) and its impurities for 24 h. **(A)** Relative swimming times during 20-min darkness. **(B)** Relative average speeds during 20-min darkness. **(C–E)** Sample movement tracking plots for experiments with CTX, impurity A, and 7-aminocephalosporanic acid. These images show the tracking of the fish (green tracks) over a 2-min period, not the total 20-min locomotion tracks during the experiment. Each well in any given row contained the same compound. Data are expressed as the mean ± SEM of three replications (eight larvae per replication). Results were evaluated using an ANOVA analysis to independently establish if treatments statistically reduced or increased the locomotor behavior in comparison to the control group (the level of statistical significance is indicated as ^∗^*p* < 0.05, ^∗∗^*p* < 0.01, and ^∗∗∗^*p* < 0.001).

The free-swimming times and speeds of zebrafish after exposure to CTX and five impurities (A, C, E, 7-ACA, and ATMAA) during 20 min of continuous darkness are shown in **Figure 2**. For CTX, the swimming time of the exposed larvae tended to decline in a concentration-dependent manner, with a significant decrease at 20 and 30 mM compared with the control. For 7-ACA, the swimming time was also significantly decreased at the highest concentration. The swimming times were not affected in the impurity A, C, E, or ATMAA treatment groups. As shown in **Figure 2B**, the average swimming speeds were not significantly affected by CTX, impurities C or E, or ATMAA treatment. The average swimming speeds were significantly decreased in the low-concentration (1 and 5 mM) impurity A-treated groups and significantly increased in the low-concentration (10 mM) 7-ACA-treated group.

**FIGURE 3:**
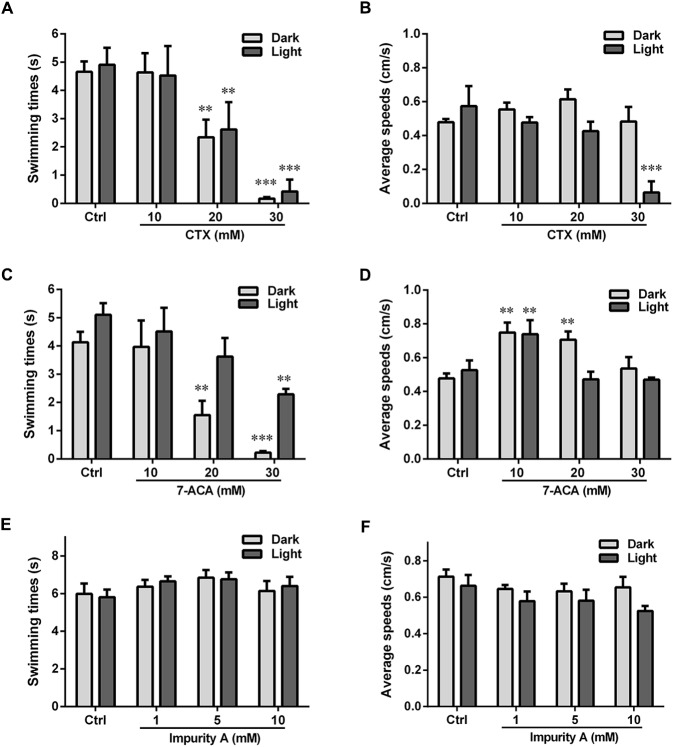
Locomotor behavior of the larval zebrafish after exposure to various concentrations of CTX and its impurities for 24 h with the dark–light–dark photoperiod stimulation. **(A,C,E)** Relative swimming times with the photoperiod stimulation. **(B,D,F)** Relative average speeds with the photoperiod stimulation. Data are expressed as the mean ± SEM of three replications (eight larvae per replication). Results were evaluated using an ANOVA analysis to independently establish if treatments statistically reduced or increased the locomotor behavior in comparison to the control group (the level of statistical significance is indicated as ^∗^*p* < 0.05, ^∗∗^*p* < 0.01, and ^∗∗∗^*p* < 0.001).

We also measured the locomotor activity with the alternating dark–light–dark photoperiod stimulation (**Figure 3**). For CTX and 7-ACA, the swimming time exhibited a similar downward tendency: with increasing chemical concentration for each photoperiod, the swimming time in the dark period was much lower than that in the control group (**Figures 3A,C**). However, CTX treatment did not influence the average swimming speed of the larvae, with the exception of the 30-mM CTX-treated group, which had lower swimming speeds than the control group during the light period (**Figure 3B**). Exposure to 7-ACA at the lowest concentration resulted in a significant increasing trend in average swimming speed in every photoperiod (**Figure 3D**). The swimming times and speeds were not affected by impurity A, C, E, or ATMAA treatment (data for impurities C, E, and ATMAA are not shown here).

ATMAA, the intermediate of the synthesis of CTX, had no effect on zebrafish behavior, suggesting that the aminothiazoyl ring at the C-7 position may not be responsible for the behavioral response effects in the larvae. Furthermore, the changes in the swimming times and speeds indicated that exposure to CTX and its mother nucleus structure 7-ACA influenced the motor nerve system development of the larvae. Impurity A is different from CTX at the C-3 side chain, and the larvae’s behavioral responses to these two compounds were also different. Impurities C and E, which differ from CTX at the C-7 side chain and C-3 side chain, respectively, did not induce behavioral alterations. These results suggest that 7-ACA is the key neurotoxic functional group of CTX, and the structure of the C-3 side chain also affects the motor nervous system of zebrafish larvae.

### Effects of Cephalosporins on the Behavior of Larval Zebrafish

**FIGURE 4:**
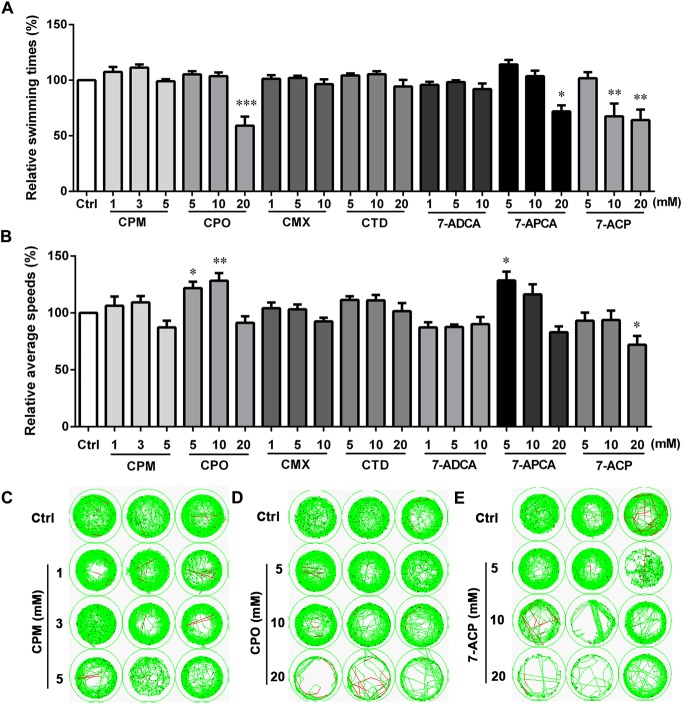
Locomotor behavior of the larval zebrafish after exposure to various concentrations of cephalosporin for 24 h. **(A)** Relative swimming times during 20-min darkness. **(B)** Relative average speeds during 20-min darkness. **(C–E)** Sample movement tracking plots for experiments with cefepime (CPM), cefpirome (CPO), and (6R,7R)-7-amino-3-(6,7-dihydro-5H-cyclopenta[b]pyridin-1-ium-1-ylmethyl)-8-oxo-5-thia-1-azabicyclo[4.2.0]oct-2-ene-2-carboxylate (7-ACP). These images show the tracking of the fish (green tracks) over a 2-min period, not the total 20-min locomotion tracks during the experiment. Each well in any given row contained the same compound. Data are expressed as the mean ± SEM of three replications (eight larvae per replication). Results were evaluated using an ANOVA analysis to independently establish if treatments statistically reduced or increased the locomotor behavior in comparison to the control group (level of statistical significance is indicated as ^∗^*p* < 0.05, ^∗∗^*p* < 0.01, and ^∗∗∗^*p* < 0.001).

The locomotion activities of larvae after exposure to CPM, CPO, CTD, CMX, 7-ADCA, 7-APCA, and 7-ACP for 24 h were further assessed under continuous darkness (**Figure 4**). CPO showed statistically significant movement speed increases at 5 and 10 mM and statistically significant movement times’ suppression at 20 mM compared with the control. At a high concentration, 7-ACP significantly decreased the swimming times and speeds. The 7-APCA group showed a significant movement speed increase at 5 mM and significant movement suppression at 20 mM compared with the control. As shown in **Figure 4**, the average swimming times and speeds were not affected by CPM, 7-ADCA, CTD, or CMX at any treatment concentration. The changes in the swimming times and speeds in these groups indicated that the structure of the C-3 side chain affects the motor nervous system of zebrafish larvae.

### Transcriptional Response to Behavioral Alterations in Zebrafish

To detect the whole embryo transcriptional profiles of the zebrafish, RNAs generated from control zebrafish embryos and embryos exposed to the six compounds were individually hybridized using the Agilent Zebrafish Microarray analysis kit. We screened a total of 45,723 transcripts for genes with fold changes (FCs) ≥ 3 or < 0.33. Then, GO (biological process enrichment) analyses identified DEGs that were associated with nervous system and sensory organ development. As shown in **Table 2**, the CTX group significantly regulated six biological processes: neuron part, response to light stimulus, neurological system process, regulation of neurotransmitter levels, sensory perception, and synapse. ATMAA significantly regulated six biological processes: sensory perception, neurological system process, central nervous system development, visual perception, response to light stimulus, and sensory perception of light stimulus. Impurity A significantly regulated six biological processes: neuron part, synaptic signaling, nervous system development, neurological system process, sensory perception, and modulation of synaptic transmission. CMX significantly regulated eight biological processes: neuron part, sensory perception, neurological system process, synapse, cellular response to light stimulus, phototransduction, detection of light stimulus, and behavior. CPO significantly regulated nine biological processes: neuron part, response to light stimulus, neurological system process, cellular response to light stimulus, phototransduction, detection of light stimulus, sensory perception of light stimulus, sensory perception, and visual perception. CPM significantly regulated 10 biological processes: sensory perception, neurological system process, response to light stimulus, cellular response to light stimulus, feeding behavior, detection of light stimulus, visual perception, sensory perception of light stimulus phototransduction, and behavior.

**Table 2:** List of identified genes related to nervous system and sensory organ development that were significantly differently expressed in zebrafish embryos treated with different chemicals.

Enriched biological process	CTX	ATMAA	Impurity A	CMX	CPO	CPM
Neuron part	*opn1mw3*		*grm1a*	*opn7c*	*opn8c*	
	*rhol*		*nsmfb*	*opn8c*	*pcdh15b*	
	*snap25a*		*opn7c*	*cpe*	*slc6a2*	
	*snap25b*		*opn8c*	*grm1a*	*grm1a*	
	*synpr*		*oprl1*		*drd2a*	
			*slc18a3a*		*drd2a*	
			*sypa*		*cpe*	
Neurological system process	*opn1mw3*	*vsx1*	*cryaa*	*or111-4*	*or111-5*	*or111-4*
	*or106-4*	*opn1mw2*	*dfnb31a*	*or111-52*	*>pcdh15b*	*or111-5*
	*or111-4*	*dfnb31a*	*opn7c*	*opn7c*	*>tmtops3a*	*rgra*
	*rhol*	*or125-6*	*oprl1*	*or118-3*	*arr3a*	*pou4f3*
			*or111-5*	*or126-3*	*or118-3*	*or125-6*
			*or113-2*		*opn3*	*slc17a6b*
			*or118-3*			*opn3*
			*or126-3*			*opn3*
			*tas2r202*			*rpe65a*
						*or113-2*
						*or126-3*
						*or127-1*
Regulation of neurotransmitter levels	*gad2*					
	*snap25b*					
Synaptic signaling			*slc18a3a*			
			*oprl1*			
			*sypa*			
			*grm1a*			
			*rapsn*			
			*nsmfb*			
Response to light stimulus	*opn1mw3*	*nr3c1*			*opn8c*	*opn8c*
	*rcvrna*	*opn1mw2*			*rcvrna*	*opn8c*
	*rhol*	NA			*opn6b*	*tp73*
	*rhol*	NA			*pcdh15b*	*rgra*
					*tmtops3a*	*opn3*
					*opn3*	*nr3c1*
					*opn3*	*nr3c1*
Sensory perception	*opn1mw3*	*vsx1*	*cryaa*	*or111-4*	*or111-5*	*or111-4*
			*or111-5*	*or126-3*	*or118-3*	*or125-6*
			*or113-2*	*or111-4*	*or111-5*	*or111-4*
			*or118-3*	*or126-3*	*or118-3*	*or125-6*
			*or126-3*	*or113-2*	*oopn3*
			*tas2r202*		*or113-2*	*or118-3*
						*opn3*
						*rpe65a*
						*or113-2*
						*or126-3*
						*or127-1*
	*or106-4*	*opn1mw2*	*dfnb31a*	*or111-5grm1a*	*pcdh15b*	*or111-5*
	*or111-4*	*dfnb31a*	*opn7c*	*opn7cgrm1a*	*tmtops3a*	*rgra*
	*rhol*	*or125-6*	*oprl1*	*or118-3grm1a*	*arr3a*	*pou4f3*
			*or111-5*	*or126-3*	*or118-3*	*or125-6*
			*or113-2*	*or113-2*	*opn3*	*slc17a6b*
			or118-3			or118-3
			or126-3			opn3
			tas2r202			rpe65a
						or113-2
						or126-3
						or127-1
Synapse	*snap25a*			*grm1a*		
	*snap25b*			*gabrr1*		
	*synpr*			*cpe*		
	*vwc2l*			*gabrr3a*		
Cellular response to light stimulus				*opn7*	*tmtops3a*	*tp73*
				*opn8c*	*opn8c*	*opn8c*
				NA	*opn3*	*opn3*
Detection of light stimulus				*opn7c*	*tmtops3a*	*opn8c*
				*opn8c*	*opn6b*	*opn3*
				NA	*opn8c*	*rgra*
					*pcdh15b*	
					*opn3*	
Phototransduction				*opn7c*	*tmtops3a*	*opn8c*
				*opn8c*	*opn6b*	*opn3*
				NA	*opn8c*	*rgra*
					*opn3*	
Sensory perception of light stimulus		*opn1mw2*			*tmtops3a*	*opn3*
		*dfnb31a*			*arr3a*	*rpe65a*
		*vsx1*			*pcdh15b*	*slc17a6b*
					*opn3*	*rgra*
Visual perception		*opn1mw2*			*tmtops3a*	*opn3*
		*vsx1*			*arr3a*	*rpe65a*
		NA			*pcdh15b*	*slc17a6b*
					*opn3*	*rgra*
Feeding behavior						*pyya*
						*npy4r*
						*cart2*
Central nervous system development		*vsx1*				
		*fgf22*				
		*olig1*				
		*hapln4*				
		*prnprs3*				
Modulation of synaptic transmission			*grm1a*			
			*nsmfb*			
			*sypa*			
Nervous system development			*cdk5r1b*			
			*ncam1b*			
			*lhx4*			
			*rtn4r*			
			*kif17*			
			*neurod2*			
			*ecrg4a*			
			*cdc73*			
			*angpt1*			
			*nsmfb*			
			*rapsn*			
			*kif26bb*			
			*acanb*			
Behavior				*cart2*		*desmb*
				*htr2b*		*pyya*
				*NA*		*cart2*
						*nr3c1*
						*npy4r*

*NA, unknown function gene; CMX, cefmenoxime; CTX, cefotaxime; CPO, cefpirome; CPM, cefepime; ATMAA, 2-(2-aminothiazole-4-yl)-2-methoxyiminoacetic acid*.

**FIGURE 5:**
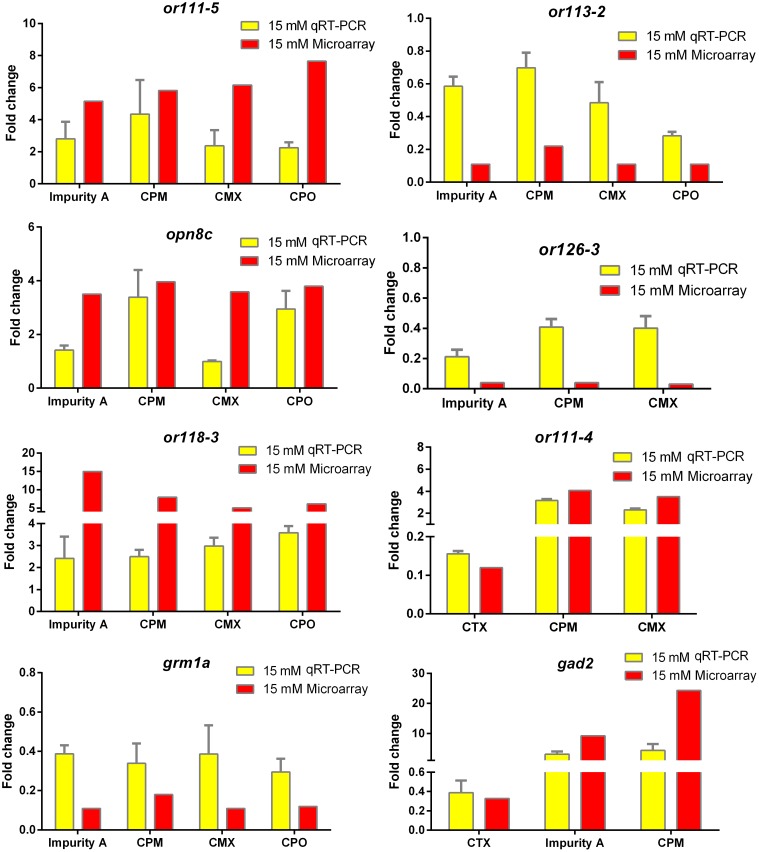
Quantitative real-time polymerase chain reaction (qRT-PCR) validation of selected genes and comparison with microarray data. Values of fold change (FC) by qRT-PCR are given as mean of FC ± standard deviation (*n* = 3 pools of 30 embryos each). Red bars represent microarray results and yellow bars represent qRT-PCR results.

Quantitative real-time polymerase chain reaction (qRT-PCR) analysis was used to confirm the eight selected DEGs that were identified by microarray analysis. The expression data for this gene set as detected by microarray and qRT-PCR analyses are plotted in **Figure 5**. Results showed that CPM, CPO, impurity A, and CMX upregulated *or111-5 and or118-3* expression, CPM and CPO inhibited *or113-2* expression, CPM and CPO upregulated *opn8c* expression, CPM, impurity A, and CMX downregulated gene *or126-3*, CTX downregulated *or111-4 and gad2* expression, CPM and CMX upregulated *or111-4*, CPM, CPO, impurity A, and CMX downregulated *grm1a*, and impurity A and CPM upregulated gene *gad2*, as detected by both qRT-PCR and microarray analyses. These results thereby confirm the reliability of our microarray analysis method.

CTX significantly regulated nine genes: *or106-4*, *or111-4*, *vwc2l*, *gad2*, *opn1mw3*, *rcvrna*, *rhol*, *snap25a*, *snap25b*, and *synpr*. ATMAA significantly regulated nine genes: *fgf22*, *hapln4*, *nr3c1*, *olig1*, *or125-6*, *prnprs3*, *dfnb31a*, *opn1mw2*, and *vsx1*. Impurity A significantly regulated 26 genes: *cryaa*, *acanb*, *angpt1*, *cdc73*, *cdk5r1b*, *ecrg4a*, *kif17*, *kif26bb*, *lhx4*, *ncam1b*, *rapsn*, *rtn4r*, *dfnb31a*, *or111-5*, *or113-2*, *or118-3*, *or126-3*, *tas2r202*, *grm1a*, *neurod2*, *nsmfb*, *opn7c*, *opn8c*, *oprl1*, *slc18a3a*, and *sypa*. CMX significantly regulated 13 genes: *cart2*, *gabrr1*, *gabrr3a*, *htr2b*, *or111-4*, *or111-5*, *or113-2*, *or118-3*, *or126-3*, *cpe*, *grm1a*, *opn7c*, and *opn8c*. CPO significantly regulated 16 genes: *or111-5*, *or113-2*, *or118-3*, *arr3a*, *cpe*, *drd2a*, *grm1a*, *opn3*, *opn6b*, *opn8c*, *otpa*, *pcdh15a*, *pcdh15b*, *rcvrna*, *slc6a2*, and *tmtops3a*. CPM significantly regulated 19 genes: *npy4r*, *or111-4*, *or111-5*, *or113-2*, *or118-3*, *or125-6*, *or126-3*, *cart2*, *or127-1*, *rpe65a*, *slc17a6b*, *desmb*, *nr3c1*, *opn3*, *opn8c*, *pou4f3*, *pyya*, *rgra*, and *tp73*. At the level of the transcriptome, the responses to CTX, CPO, CPM, CMX, impurity A, and ATMAA in terms of significant changes in gene expression were different. CMX and ATMAA resulted in a lower number of regulated genes compared to the other four compounds.

We also used Venn diagrams to determine overlaps of significantly DEGs between compounds. CTX, impurity A, and CPM co-regulated one gene: *gad2*. CMX, CPM, and CTX co-regulated one gene: *or111-4*. CMX, CPM, and impurity A co-regulated one gene: *or126-3*. CMX, CPM, CPO, and impurity A co-regulated five genes: *grm1a*, *opn8c*, *or111-5*, *or113-2*, and *or118-3*.

### PPI Network Analysis of DEG Response to Behavioral Alterations

**FIGURE 6:**
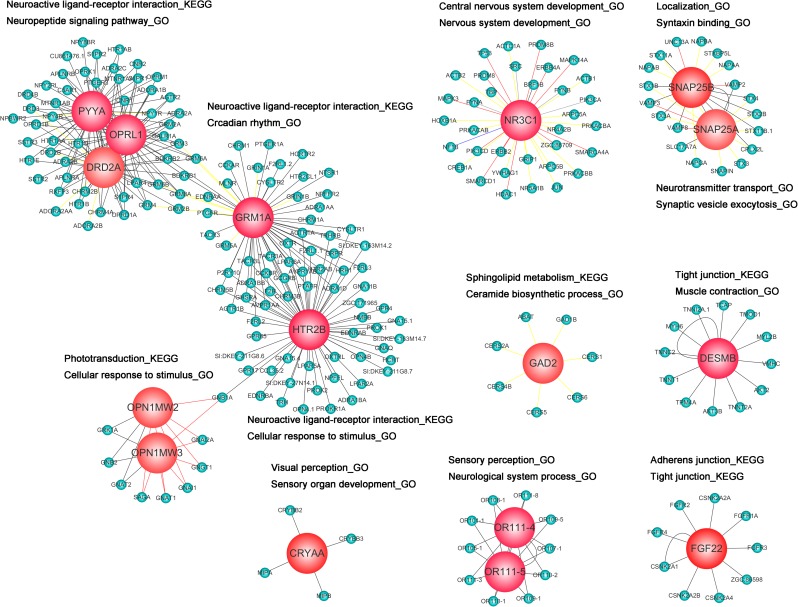
Protein–protein interaction network of differentially expressed genes (DEGs) based on the number of interactions. Light blue signifies non-DEGs and red signifies hub proteins (DEGs). Proteins were also mapped to the enriched GO terms and Kyoto Encyclopedia of Genes and Genomes pathways.

To interpret the biological meaning of the identified DEG response to behavioral alterations at the protein level, we constructed a PPI network of the proteins encoded by the DEGs, which included 245 nodes and 380 edges. From the PPI network of proteins encoded by DEGs, the 16 hub proteins—CRYAA, DESMB, FGF22, GAD2, HTR2B, OPN1MW2, OPN1MW3, OR111-4, OR111-5, DRD2A, GRM1A, NR3C1, OPRL1, PYYA, SNAP25A, and SNAP25B—were identified based on the number of interacting edges and used to establish 16 module clusters. **Figure 6** summarizes the significantly regulated pathways and GO terms of the 16 module clusters (*p* < 0.05). The 16 module clusters were also further identified by functional enrichment of the GO hierarchy for zebrafish embryo nervous system development (only the top five are shown). Among these, the CRYAA module was significantly enriched with the categories of “visual perception_GO,” “sensory perception of light stimulus_GO,” “sensory organ development_GO,” “sensory perception_GO,” and “neurological system process_GO.” The DESMB module was significantly enriched with the categories of “tight junction_KEGG,” “muscle contraction_GO,” “muscle system process_GO,” “sarcomere_GO,” and “myofibril_GO.” The FGF22 module was significantly enriched with the categories of “adherens junction_KEGG,” “wnt signaling pathway_KEGG,” “tight junction_KEGG,” and “regulation of actin cytoskeleton_KEGG.” The GAD2 module was significantly enriched with the categories of “sphingolipid metabolism_KEGG,” “metabolic pathways _KEGG,” “ceramide biosynthetic process_GO,” “ceramide metabolic process_GO,” and “sphingolipid biosynthetic process_GO.” The HTR2B module was significantly enriched with the categories of “neuroactive ligand–receptor interaction_KEGG,” “gap junction_KEGG,” “regulation of actin cytoskeleton_KEGG,” “cellular response to stimulus_GO,” and “response to stimulus_GO.” The OPN1MW2 and OPN1MW3 module was significantly enriched with the categories of “phototransduction_KEGG,” “endocytosis_KEGG,” “gap junction_KEGG,” “cellular response to stimulus_GO,” and “response to stimulus _GO.” The DRD2A module was significantly enriched with the categories “neuroactive ligand–receptor interaction_KEGG,” “anterograde trans-synaptic signaling _GO,” “synaptic signaling _GO,” “neuropeptide signaling pathway_GO,” and “locomotion_GO.” The GRM1A module was significantly enriched with the categories “neuroactive ligand–receptor interaction_KEGG,” “circadian rhythm_GO,” “neuropeptide signaling pathway_GO,” “anterograde trans-synaptic signaling_GO,” and “chemical synaptic transmission_GO.” The NR3C1 module was significantly enriched with the categories “central nervous system development_ GO,” “nervous system development_GO,” “locomotion_GO,” “cranial nerve development_GO,” and “neurogenesis_GO.” The OPRL1 module was significantly enriched with the categories “neuroactive ligand–receptor interaction_KEGG,” “synaptic signaling_GO,” “chemical synaptic transmission_GO,” “locomotion_GO,” and “neuropeptide signaling pathway_GO.” The OR111-4 and OR111-5 module was significantly enriched with the categories “sensory perception of chemical stimulus_GO,” “sensory perception_GO,” “neurological system process_GO,” “detection of chemical stimulus involved in sensory perception_GO,” and “detection of chemical stimulus_GO.” The PYYA module was significantly enriched with the categories “neuroactive ligand–receptor interaction_KEGG,” “neuropeptide binding_GO,” “anterograde trans-synaptic signaling_GO,” “neuropeptide signaling pathway_GO,” and “locomotion_GO.” The SNAP25A module was significantly enriched with the categories “neurotransmitter transport_GO,” “vesicle-mediated transport in synapse_GO,” “synaptic vesicle localization_GO,” “synaptic vesicle transport-GO,” and “synaptic vesicle exocytosis_GO.” Finally, the SNAP25B module was significantly enriched with the categories “localization_GO,” “neurotransmitter transport_GO,” “synaptic vesicle fusion to presynaptic active zone membrane_GO,” “synaptic vesicle exocytosis_GO,” and “syntaxin binding_GO.”

### Analysis of the Optimum Molecular Docking Poses of Different Ligands

**FIGURE 7:**
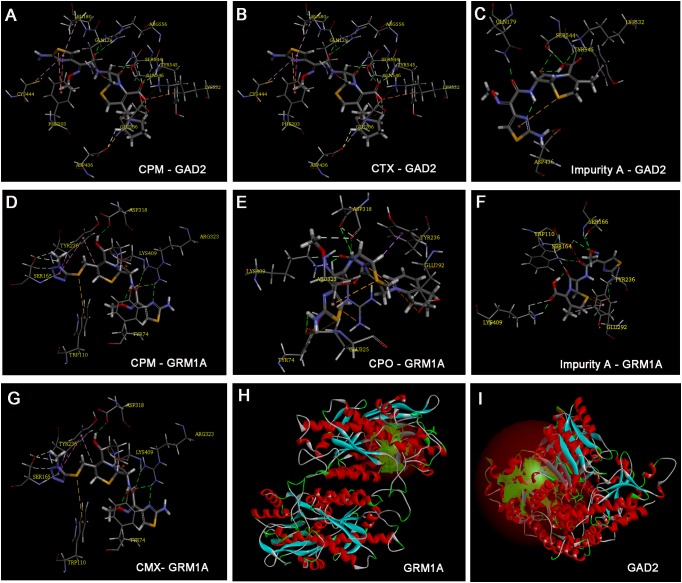
Model of ligand docking. 3D structure showing **(A)** CPM, **(B)** CTX, and **(C)** impurity A interactions with the residues of GAD2, and **(D)** CPM, **(E)** CPO, **(F)** impurity A, and **(G)** cefmenoxime interactions with the residues of glutamate receptor, metabotropic 1a (GRM1A); best docking poses of compound inside the glutamate decarboxylase 2 (GAD2) and GRM1A binding sites, showing the different interactions. Arrow: hydrogen bond formed with the side chain of the residue. **(H)** The active site of GRM1A. **(I)** The active site of GAD2.

Based on the zebrafish microarray and qRT-PCR analysis, CTX-, impurity A-, and CPM-regulated *gad2* and CPM-, CPO-, impurity A-, and CMX-regulated *grm1a*, we selected these compounds to implement the docking studies. Docking results tabulated between the GRM1A and GAD2 proteins (**Figures 7H,I**) and the compounds are shown along with the modifications within them in Supplementary Table 1. The docking results for the compounds that showed the optimum docking pose with GAD2 and GRM1A are summarized in **Figure 7**. CPM presented the formation of six hydrogen bonds (HBs) with residues of the GAD2 (**Figure 7A**). The quaternary amine group with a positive charge at the C-3 side chain and the carbonyl group of the mother nucleus of CPM restored HBs with GLU 206, LYS 532, TYR 545, SER 544, GLN 546, and ARG 556. The ligand poses with the best docking score for CTX (**Figure 7B**) presented the formation of five HBs with residues of the GAD2. In addition, the carbonyl group and the hydroxide radical at the mother nucleus of CTX formed HB interactions with the side chain of residues ARG 556, GLN 546, SER 544, and TYR 545. Impurity A presented the formation of four HBs with residues of the GAD2 (**Figure 7C**). The hydroxide radical and the carbonyl group of the mother nucleus of impurity A interacted via the formation of HBs with GLN 179, SER 544, and TYR 545. The results of the docking analysis suggested that the three compounds adopted similar binding poses in GAD2. The findings also suggest that the residues SER 544 and TYR 545 may play a vital role in the inhibition of GAD2 expression, and the C-3 side chain allows the compound to be better oriented inside the binding site.

Cefepime (CPM) presented the formation of three HBs with residues of the GRM1A (**Figure 7D**). The carboxyl group and the carbonyl group of the mother nucleus of CPM restored HBs with LYS 409 and ARG 323. CPO presented the formation of three HBs with residues of the GRM1A (**Figure 7E**). The imino group and the carbonyl group of the mother nucleus of CPO restored HBs with LYS 409 and ASP 318. The carbonyl group of the mother nucleus of impurity A presented the formation of two HBs with LYS 409 and TRP 110 residues of the GRM1A (**Figure 7F**). Furthermore, the aldoxime group and the amino group of the C-7 side chain of impurity A presented the formation of two HBs with GLU 292 and SER 166 residues of the GRM1A. The carboxyl group, the carbonyl group and the imine group of the mother nucleus of CMX presented the formation of four HBs with LYS 409, ASP318, and TYP 74 residues of the GRM1A2 (**Figure 7G**). The carbonyl group and the imine group of the C-7 side chain of CMX restored two HBs with ARG 323 residue of the GRM1A. The results implicit that the residues LYS 409 may play a vital role in the inhibition of GRM1A expression, and the C-7 side chain also combines with the binding site of some proteins. Totally, the mother nucleus 7-ACA is responsible for the compounds binds to the target proteins. In addition, the high level of identity (83.47 and 83.61%) between human and zebrafish GAD2 and GRM1A protein sequences suggests that the functions of the proteins may be related.

## Discussion

**FIGURE 8:**
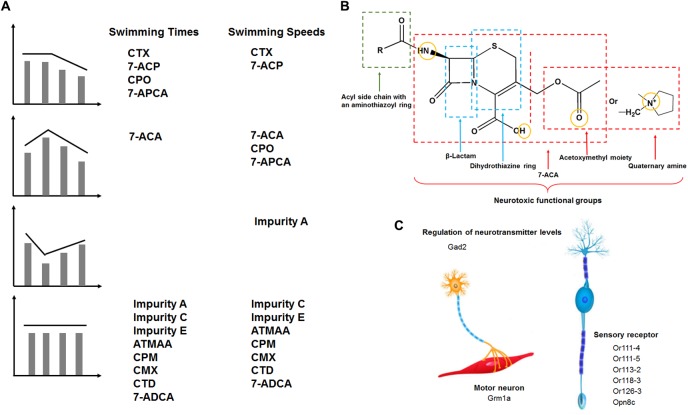
**(A)** Four categories of compound neurotoxicity manner and neurotoxic functional groups of cephalosporins. Type I: dose-dependent inhibition manner. Type II: low-concentration stimulation and high-concentration inhibition manner. Type III: low-concentration inhibition and high-concentration stimulation manner. Type IV: no dose dependence. **(B)** Schematic representation of neurotoxic functional groups of cephalosporins containing an aminothiazoyl ring at the C-7 position in zebrafish. **(C)** Drug-induced motor and sensory nervous system toxicity effects and the target genes associated with different functional regions of the motor and sensory nervous system.

Cephalosporins have the potential to induce nerve toxicological effects (Fugate et al., 2013; Stip et al., 2016). In the present study, we investigated the effects of cephalosporins with an aminothiazoyl ring at the C-7 position on the behavior of zebrafish embryos and larvae. Locomotor behaviors were assessed with free-swimming activities under continuous visible light and dark-to-light photoperiod stimulation. According to the results of the behavior tests (swimming times and speeds), we divided the compounds into four categories of neurotoxic action, as shown in **Figure 8**. In terms of swimming speeds, type I was dose-dependent inhibition (CTX and 7-ACP), type II was low-concentration stimulation and high-concentration inhibition (7-ACA, CPO, and 7-APCA), type III was low-concentration inhibition and high-concentration stimulation (impurity A), and type IV was no dose dependence (impurity C, impurity E, ATMAA, CPM, CMX, CTD, and 7-ADCA). The different neurotoxic actions of these similarly structured compounds indicate that there may be more than two pathways involved in the neurotoxicity mechanisms.

We found that exposure of zebrafish embryos to CTX decreased locomotor behavior (type I) and influenced gene expressions in regulation of neurotransmitter levels (GABA), synapse, and neuron parts, all of which are clinical characteristics of seizures arising. When treated with CTX under dark-to-light photoperiod stimulation, the larvae slowed their activity in response to the light, and disordered gene expression of the response to light stimulus and phototransduction. Interestingly, 7-ACA (the mother nucleus and C-3 side chain of CTX) displayed a low-stimulation and high-concentration inhibition manner of locomotion speeds (type II), and ATMAA (C-7 side chain of CTX) had no effects on these activity patterns in free-swimming activities under continuous darkness (type IV), influencing the genes were all different with the other chemicals. Impurity A exhibited a low-dose inhibition of locomotion speeds (type III), and the substituent at the C-3 position of impurity A was different from that of CTX. Impurity A specifically regulated the modulation of synaptic transmission. CTX and impurity A co-regulated gene *gad2*. In addition, the docking studies indicated that the carbonyl group of the mother nucleus of CTX and impurity A plays an essential role in regulating *gad2* expression, and the C-7 side chain does not respond to this effect. Moreover, CTX and impurity A separately regulated their specific DEGs, which may be related to the different substituents at the C-3 position. Together, these results suggested that the C-3 side chain and mother nucleus participate in CTX-induced motor nervous system toxicity by inhibiting *gad2* expression to affect GABA biosynthesis and sphingolipid metabolism and regulating other genes’ expression to affect neuron parts. The findings also suggest that mother nucleus (7-ACA) is responsible for the neurobehavioral changes of CTX, and the structure of the C-3 side chain also affects the behavior of zebrafish larvae.

Next, we identified the drugs (CMX, CPO, CPM, and CTD) and impurities (7-APCA, 7-ACP, and 7-ADCA). Each of these drugs also contains an aminothiazoyl structure and is related to CTX. Administration of these drugs had different effects on swimming activity patterns in dark conditions. The modes of CPO and 7-ACP involvement in locomotor behaviors were slightly different with respect to swimming speeds: CPO displayed a dose–response relationship characterized by low-dose stimulation and high-dose inhibition (type II), while 7-ACP showed high-dose inhibition (type I). As 7-ACP contains both the mother nucleus and C-3 substituent of CPO, this result indicated that the mother nucleus and C-3 substituent structure plays a key role in CPO neurotoxicity. CPM structure differs from CPO at the C-3 substituent. In our study, CPM had no effects on zebrafish larvae behaviors at any treatment dosage, which means that CPO causes more obvious adverse neurologic effects than CPM in zebrafish larvae. However, transcriptomics analysis showed that CPO and CPM co-regulated five DEGs, which may be driven by the same C-7 side chain and mother nucleus structure of CPO and CPM. The two drugs separately regulated their specific DEGs, which may be driven by the different C-3 substituent. The docking studies showed that the C-3 substituent and mother nucleus structure of CPM could work together in regulating *gad2* and *grm1a* expression to induce neurotoxicity. 7-APCA is an important intermediate in the synthesis process of CTD; specifically, CTD is the product after modification of the C-3 and C-7 substituents of 7-APCA. 7-APCA had a dose-response effect characterized by low-dose stimulation and high-dose inhibition (type II). However, CTD did not alter the general response pattern in zebrafish larvae, which suggested that the modification of the C-3 and C-7 substituents of 7-APCA could inhibited its neurotoxicity. In sum, our findings suggest that the mother nucleus and C-3 substituent play key roles in the regulation of this class of cephalosporin neurotoxicity.

Recent research has indicated that the adverse effects of CPM and CTD include neurotoxicity, with symptoms such as seizures, confusion, delirium, and myoclonus, particularly when CPM is administered at high doses in patients with renal failure (Honore and Spapen, 2015; Mani et al., 2015; Lindsay et al., 2017). Many studies have suggested that the mechanism of CPM neurotoxicity consists of the inhibition of GABA release at the GABA-A receptor and the disruption of inhibitory synaptic transmission, increased glutamate, and cytokine release (Chow et al., 2005; Gangireddy et al., 2011; Lindsay et al., 2017). We found that CPM and CPO may play roles in sensory and motor nervous system injury to zebrafish larvae. This finding suggests that CPM may interfere with neurodevelopment and alter perception and behavior not only through neuroactive ligand–receptor interaction, enkephalin release, and the metabotropic glutamate receptor group II pathway, as found in previous studies, but also through circadian rhythm, neuropeptide signaling pathway, and sphingolipid metabolism. For the drugs tested in this study, CPM did not significantly alter larval swimming behavior. However, CPM significantly regulated genes and pathways connected to the nervous system. These findings indicate that behavior testing may be adversely affected by neurotoxicity in developing zebrafish. However, the use of pathways as well as protein module clusters may be useful in identifying gene biomarkers for predicting the neurotoxicity of compounds in zebrafish embryos.

Taken together, the results of our investigation on zebrafish larvae behavioral detection suggest that the neurotoxicity of these types of similarly structured drugs is dependent on the 7-ACA mother nucleus, and the substituent at the C-3 position also plays a role in altering nervous system function (**Figures 8B,C**). It is noteworthy that the drugs that have a quaternary amine group with a positive charge at the C-3 position (e.g., CPO, CTD, and CPM) more strongly inhibit the neuroactive ligand–receptor interaction pathway. We hypothesize that cephalosporin with an aminothiazoyl ring at the C-7 position may induce a mixed pattern of gene transcription through the neuroactive ligand–receptor interaction (e.g., GABA), and sphingolipid metabolism in specific regions of the sensory and motor nervous system in zebrafish larvae, all of which are characteristics of neurological disorders in humans. Overall, our study established a model in 5 dpf zebrafish larvae and demonstrated its utilization in identifying the relationship between the drug structure and neurotoxicity, which is one of the key requirements of drug quality control. Future studies will aim to elucidate the neurotoxicity mechanisms of these drugs and impurities.

## Author Contributions

JZ and CH conceived, designed, and supervised the study. YH performed the transcriptome and docking experiments and analyzed the data. YH and CH wrote the manuscript. YZ performed the zebrafish behavior test.

## Conflict of Interest Statement

The authors declare that the research was conducted in the absence of any commercial or financial relationships that could be construed as a potential conflict of interest.

## Footnotes

1 http://oacu.od.nih.gov/regs/index.htm

2 http://bioinfogp.cnb.csic.es/tools/venny/index.html

3 http://thebiogrid.org/

4 http://string-db.org/

5 http://www.cytoscape.org/

6 https://swissmodel.expasy.org/

7 https://www.ncbi.nlm.nih.gov/pccompound/
