# Supplementary material for: Neurobehavioral Effects of Cephalosporins: Assessment of Locomotors Activity, Motor and Sensory Development in Zebrafish
Source: Front Pharmacol. 2018 Mar 2;9:160. doi: 10.3389/fphar.2018.00160 (PMC5840155; doi:10.3389/fphar.2018.00160)
Supplement: Supplementary file 1 [file Table_1.DOCX]

**Table 1** Dock results

| Protein | Compound | -CDOCKER Energy | -CDOCKER Interaction Energy |
| --- | --- | --- | --- |
| GAD2 | CTX | 16.7454 | 50.9851 |
| GAD2 | CPM | -8.39579 | 61.5697 |
| GAD2 | Impurity A | 7.52582 | 43.5335 |
| GRM1A | CPO | 3.66549 | 39.9126 |
| GRM1A | CMX | -31.7165 | 46.8547 |
| GRM1A | CPM | -9.51797 | 51.9897 |
| GRM1A | Impurity A | 3.66549 | 39.9126 |
